# Interfacial Reconstruction Enables Enhanced Thermoelectric Performance in Bismuth Telluride‐Based Alloys

**DOI:** 10.1002/advs.78266

**Published:** 2026-10-15

**Authors:** Wenyi Chen, Xiao‐Lei Shi, Meng Li, Chao Zhang, Konstantin Faershteyn, Siqi Liu, Yongqi Chen, Chengjiong He, Wanyu Lyu, Jun Pei, Nanhai Li, Zhi‐Gang Chen

**Affiliations:** ^1^ ARC Research Hub in Zero‐Emission Power Generation for Carbon Neutrality Centre for Materials Science, and School of Chemistry and Physics Queensland University of Technology Brisbane Queensland Australia; ^2^ Central Analytical Research Facility Institute for Future Environments Queensland University of Technology Brisbane Queensland Australia

**Keywords:** Bi_0.4_Sb_1.6_Te_3_, device, interfacial engineering, SnSe, thermoelectric

## Abstract

Bismuth telluride‐based alloys are the benchmark thermoelectric materials for near‐room‐temperature power generation and solid‐state cooling, yet further performance improvements through conventional optimization strategies remain challenging. Here, we introduce an interfacial reconstruction strategy by incorporating SnSe into Bi_0.4_Sb_1.6_Te_3_. Interdiffusion and chemical reconstruction at the Bi_0.4_Sb_1.6_Te_3_/SnSe heterointerfaces synergistically regulate charge‐carrier transport and phonon scattering. As a result, the optimized composite achieves a peak dimensionless figure of merit (*ZT*) of ∼1.38 at 363 K and an average *ZT* of ∼1.25 across 303–423 K. Moreover, a single‐leg thermoelectric device fabricated from this material delivers an energy conversion efficiency of ∼6.47% under a temperature difference of 200 K. These results establish interfacial reconstruction as a simple and effective approach to improving the thermoelectric performance of bismuth telluride‐based alloys.

## Graphical abstract

Bismuth telluride‐based alloys are benchmark thermoelectric materials for near‐room‐temperature applications. Here, SnSe‐induced interfacial reconstruction simultaneously optimizes carrier and phonon transport in Bi_0.4_Sb_1.6_Te_3_, achieving a peak *ZT* of ∼1.38 at 363 K and a device efficiency of ∼6.47%, providing an effective strategy for high‐performance thermoelectrics.

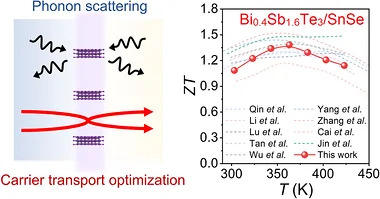

## 1 Introduction

Thermoelectric technology has attracted considerable attention as a sustainable energy‐harvesting solution for emerging artificial intelligence (AI) systems, where high‐performance AI accelerators generate substantial amounts of waste heat [1, 2, 3]. By directly converting thermal energy into electricity, thermoelectric devices (TEDs) provide an attractive route for harvesting this otherwise dissipated energy [3, 4]. Moreover, TEDs offer several intrinsic advantages, including environmentally benign operation, the absence of moving parts and working fluids, and exceptional reliability, making them well suited for long‐term, maintenance‐free operation in compact electronic systems [5, 6]. The energy conversion efficiency (*η*) of TEDs is fundamentally governed by the thermoelectric performance of their constituent materials, which is quantified by the dimensionless figure of merit, *ZT*, defined as *ZT* = *S* ^2^ *σT*/*κ*, where *S* is the Seebeck coefficient, *σ* is the electrical conductivity, *T* is the absolute temperature, and *κ* is the total thermal conductivity comprising electronic (*κ*_e_) and lattice (*κ*_l_) contributions [7, 8]. However, further improving *ZT* remains a longstanding challenge because these transport parameters are intrinsically coupled, and optimizing one often compromises the others [9, 10]. Therefore, developing effective strategies to decouple these interdependent transport properties and achieve their synergistic optimization is critical for advancing high‐performance thermoelectric materials [11, 12].

Among the various thermoelectric materials, Bi_2_Te_3_‐based alloys are widely recognized as the benchmark materials for near‐room‐temperature power generation and solid‐state cooling owing to their outstanding thermoelectric performance [5, 13]. These materials are generally classified into n‐type Bi_2_Te_3−_ *_x_* Se*_x_* and p‐type Bi*_x_* Sb_2−_ *_x_* Te_3_ alloys [5, 14]. For p‐type Bi*_x_* Sb_2−_ *_x_* Te_3_, partial substitution of Bi for Sb effectively optimizes the carrier concentration (*n*) while introducing lattice distortion, thereby suppressing *κ*_l_ through enhanced phonon scattering without compromising electrical transport [2, 5]. Consequently, compositions such as Bi_0.4_Sb_1.6_Te_3_ and Bi_0.5_Sb_1.5_Te_3_ exhibit excellent thermoelectric performance and have become the dominant p‐type materials in near‐room‐temperature TEDs [2, 15]. Over the past decades, extensive efforts have been devoted to further improving the thermoelectric performance of p‐type Bi*_x_* Sb_2−_ *_x_* Te_3_ through elemental doping with Mg [16], Mn [17], Cu [18], Ag [19], Zn [20], and other elements. These doping strategies effectively optimize *n* and modulate the electronic structure; however, their effectiveness has gradually approached a performance ceiling because of the intrinsic coupling among thermoelectric transport parameters [2, 21]. To overcome this limitation, the introduction of secondary phases has emerged as an effective strategy for simultaneously optimizing electrical and thermal transport [22, 23, 24]. A variety of inclusions, including SiC nanoparticles [25], NaBiS_2_ [26], carbon microfibers [27], Fe_3_O_4_ [28], Ag_8_GeTe_6_ [29], and MgB_2_ [30] have been incorporated into Bi*_x_* Sb_2−_ *_x_* Te_3_ matrices to tailor charge‐carrier and phonon transport synergistically. Notably, Ag_8_GeTe_6_/(Bi, Sb)_2_Te_3_ composites have achieved a peak *ZT* of 1.53 near room temperature, highlighting the considerable potential of inclusion engineering for developing high‐performance thermoelectric materials [29].

Bi*_x_* Sb_2−_ *_x_* Te_3_ alloys have been synthesized using a variety of approaches, including ball milling [31, 32, 33, 34], zone melting [32, 35], and hydrothermal/solvothermal methods [36]. Among these techniques, solvothermal synthesis is particularly attractive because it enables precise control over product composition through adjustment of precursor stoichiometry, while the reaction kinetics and thermodynamics can be readily tailored by varying the pH, temperature, and pressure [2, 36]. Moreover, solvothermal synthesis offers excellent control over the particle size and morphology of Bi*_x_* Sb_2−_ *_x_* Te_3_ powders, thereby facilitating the formation of highly anisotropic microstructures that are favorable for enhancing carrier mobility (*µ*) [36]. Despite these advantages, systematic investigations into the microstructural evolution and incorporation mechanisms of solvothermally synthesized Bi*_x_* Sb_2−_ *_x_* Te_3_ remain scarce [36, 37]. This limited understanding has hindered the broader application of solvothermal synthesis in high‐performance thermoelectric materials [37, 38]. In parallel, although secondary‐phase engineering has significantly advanced the performance of Bi*_x_* Sb_2−_ *_x_* Te_3_‐based thermoelectric materials, most reported inclusions function primarily as passive scattering centers that either enhance phonon scattering or locally modify carrier transport [22, 26, 29]. Their potential to actively reconstruct interfacial chemistry, promote elemental diffusion, and regulate local phase evolution has received little attention. Therefore, developing interfacial reconstruction agents capable of inducing favorable chemical and structural evolution represents a promising strategy for simultaneously optimizing carrier and phonon transport, thereby achieving further improvements in thermoelectric performance.

Motivated by these considerations, SnSe was selected as the interfacial reconstruction agent because its layered chalcogenide structure and chemical compatibility with Bi‐Sb‐Te‐based compounds facilitate elemental redistribution and interfacial reaction during thermal processing. Unlike conventional inert inclusion, SnSe therefore actively participates in interfacial chemical reconstruction. An “interfacial reconstruction agent” refers to a secondary component that actively induces elemental redistribution, chemical mixing, and structural reconstruction at the interface during processing, rather than merely serving as a passive inclusion or phonon‐scattering phase. In this work, we first synthesized plate‐like Bi_0.4_Sb_1.6_Te_3_ powders by optimizing the reactant composition and then introduced SnSe into Bi_0.4_Sb_1.6_Te_3_ as an interfacial reconstruction agent to actively engineer the interfacial chemistry and transport characteristics of the composite system. During spark plasma sintering (SPS), SnSe promotes elemental interdiffusion and chemical reconstruction at the Bi_0.4_Sb_1.6_Te_3_/SnSe interface, accompanied by the in situ formation of abundant Sb‐rich precipitates within the matrix, as illustrated in Figure 1a. The reconstructed interfaces, together with the Sb‐rich precipitates, synergistically facilitate carrier transport while generating lattice distortion and multiscale phonon‐scattering centers, thereby simultaneously enhancing electrical transport and suppressing *κ*_l_ (Figure 1b). Consequently, the optimized Bi_0.4_Sb_1.6_Te_3_ + 0.5 wt.% SnSe composite exhibits an ultralow *κ*_l_ of 0.40 W m^−1^ K^−1^ and achieves a competitive peak *ZT* of ∼1.38 at 363 K, outperforming most previously reported Bi*_x_* Sb_2−_ *_x_* Te_3_‐based materials (Figure 1c) [27, 29, 36, 39, 40, 41, 42, 43, 44]. Furthermore, finite‐element simulations and experimental device evaluation demonstrate that a single‐leg TED fabricated from the optimized material delivers a *η* of 6.47% under a temperature difference (Δ*T*) of 200 K (Figure 1d) [26, 29, 41, 42, 44, 45, 46, 47, 48]. These findings establish interfacial reconstruction as an effective strategy for the synergistic regulation of carrier and phonon transport, offering a general design paradigm for developing high‐performance thermoelectric materials and energy‐conversion devices.

**FIGURE 1:**
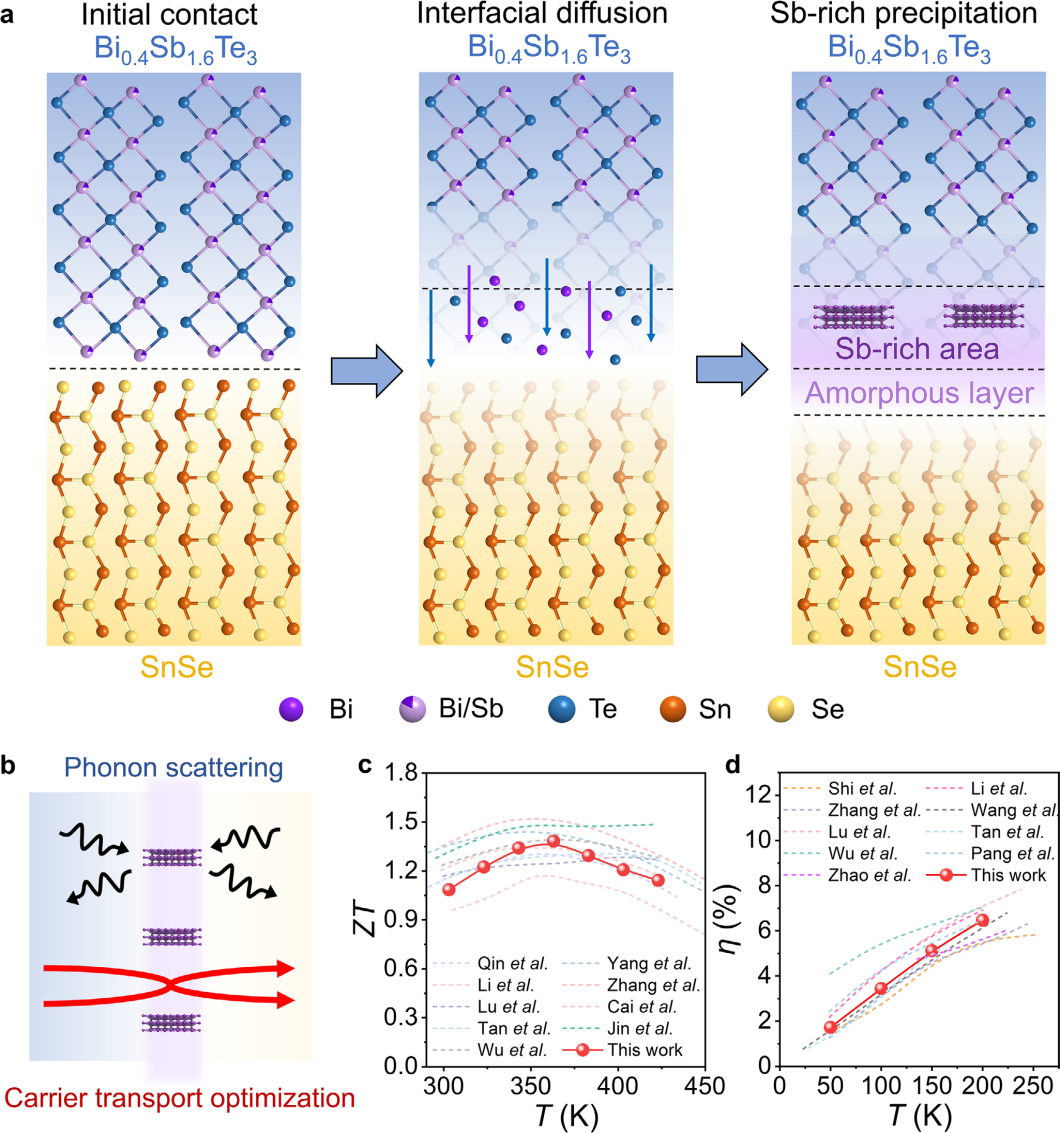
SnSe‐induced interfacial reconstruction for enhanced thermoelectric performance of Bi_0.4_Sb_1.6_Te_3_. (a) Schematic illustration of the interfacial reconstruction mechanism at the Bi_0.4_Sb_1.6_Te_3_/SnSe interface. (b) Schematic illustration of the synergistic enhancement of carrier transport and phonon scattering induced by interfacial reconstruction. (c) Comparison of the temperature‐dependent figure of merit (*ZT*) of the optimized Bi_0.4_Sb_1.6_Te_3_ + 0.5 wt.% SnSe composite with previously reported Bi_0.4_Sb_1.6_Te_3_‐based materials [27, 29, 36, 39, 40, 41, 42, 43, 44]. (d) Comparison of the energy conversion efficiency (*η*) of the fabricated thermoelectric device (TED) with previously reported Bi_2_Te_3_‐based TEDs [26, 29, 41, 42, 44, 45, 46, 47, 48].

## 2 Results and Discussion

### 2.1 Microstructure of Bi_0.4_Sb_1.6_Te_3_ Incorporated With SnSe

To systematically investigate the effect of SnSe incorporation, a series of Bi_0.4_Sb_1.6_Te_3_ + *x* wt.% SnSe composites (*x* = 0, 0.25, 0.5, 0.75, 1, and 1.5) were synthesized via a solvothermal‐assisted route followed by SPS. The phase compositions of the obtained samples were first characterized by X‐ray diffraction (XRD). As shown in Figure 2a, all major diffraction peaks can be indexed to the rhombohedral Bi_0.4_Sb_1.6_Te_3_ phase (PDF #03‐065‐3674, space group *R* $\bar{3}$ *m*), indicating that SnSe incorporation does not alter the primary crystal structure of the matrix. No discernible diffraction peaks corresponding to SnSe are detected, which can be attributed to its low content and the limited detection sensitivity of conventional XRD. To gain further insight into the structural evolution induced by SnSe incorporation, Rietveld refinement was performed for all samples. Representative refinement results for pristine Bi_0.4_Sb_1.6_Te_3_ and Bi_0.4_Sb_1.6_Te_3_ + 0.5 wt.% SnSe are shown in Figure 2b,c, respectively. The refined patterns agree well with the experimental data, with both weighted profile R‐factor (*R*_wp_) and profile R‐factor (*R*_p_) values below 10%, confirming the reliability of the structural models. The refinement results for the remaining compositions are presented in Figure S1. As can be seen, all lattice parameters gradually decrease with increasing SnSe content, indicating that SnSe incorporation induces lattice contraction accompanied by compositional redistribution within the Bi_0.4_Sb_1.6_Te_3_ matrix. The structural evolution revealed by Rietveld refinement is consistent with the subsequent microscopic observations. Figure 2d,e shows the backscattered electron (BSE) images and corresponding energy‐dispersive X‐ray spectroscopy (EDS) elemental maps of pristine Bi_0.4_Sb_1.6_Te_3_ and Bi_0.4_Sb_1.6_Te_3_ + 0.5 wt.% SnSe, respectively. Compared with the pristine sample, distinct Sb‐rich precipitates are observed in the SnSe‐containing sample, indicating that SnSe incorporation promotes local compositional redistribution and the formation of Sb‐rich regions. SEM images of the remaining compositions (0.25, 0.75, 1, and 1.5 wt.% SnSe; Figures S2–S5) show that the intensity of Sb‐rich precipitates increases progressively with increasing SnSe content. These results suggest that SnSe promotes interfacial elemental diffusion and chemical reconstruction during sintering, driving compositional redistribution within the matrix and consequently facilitating the in situ formation of Sb‐rich precipitates. The implications of this interfacial reconstruction for the microstructure and thermoelectric transport properties are discussed in the following sections.

**FIGURE 2:**
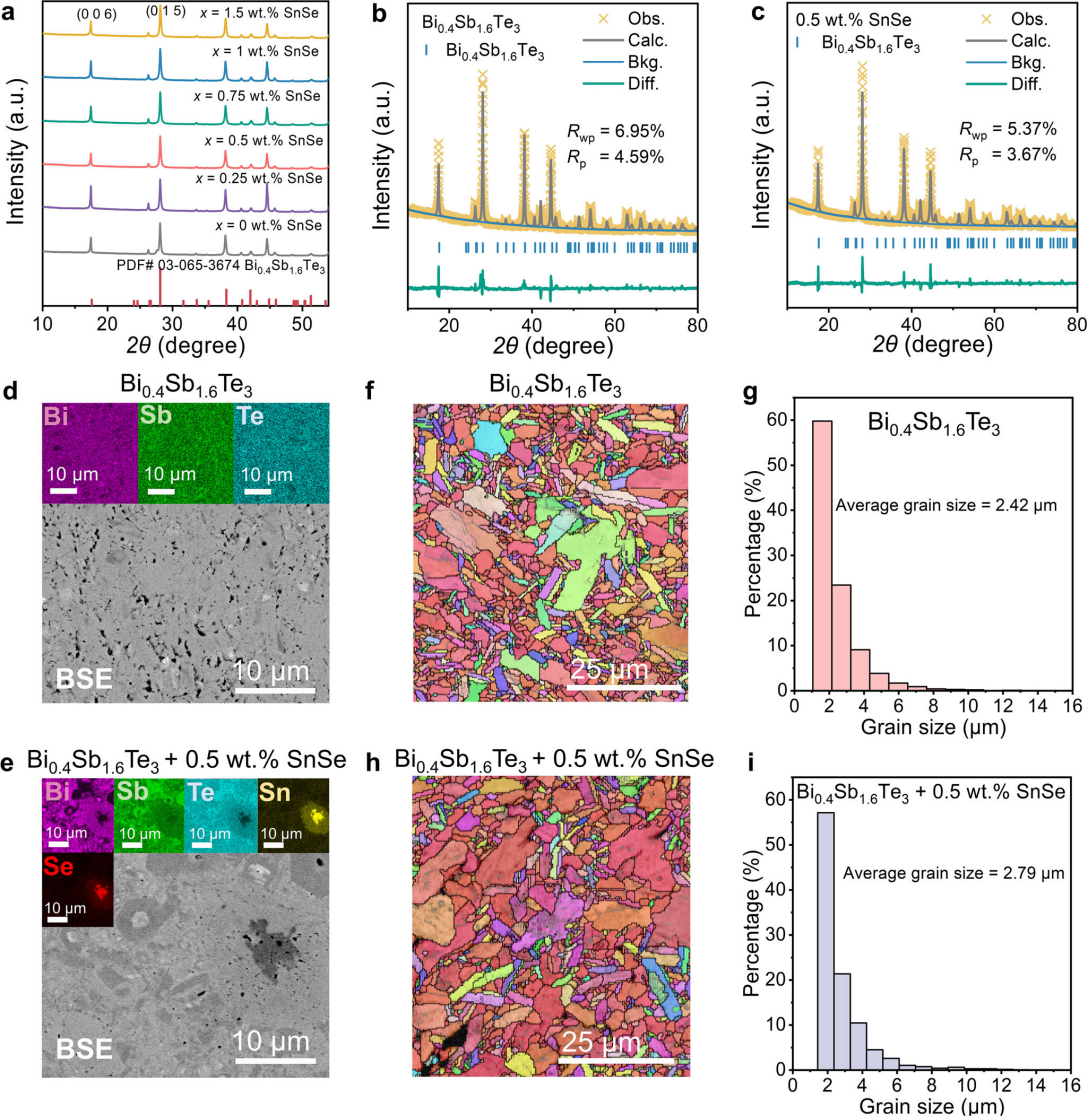
Phase and microstructural characterization of Bi_0.4_Sb_1.6_Te_3_ with SnSe‐induced interfacial reconstruction. (a) Room‐temperature X‐ray diffraction (XRD) patterns of Bi_0.4_Sb_1.6_Te_3_ + *x* wt.% SnSe (*x* = 0, 0.25, 0.5, 0.75, 1, and 1.5). Rietveld refinement of (b) pristine Bi_0.4_Sb_1.6_Te_3_ and (c) Bi_0.4_Sb_1.6_Te_3_ + 0.5 wt.% SnSe. Inset: *R*_wp_ is weighted profile R‐factor and *R*_p_ is profile R‐factor. Scanning electron microscopy (SEM) backscattered electron (BSE) images of (d) pristine Bi_0.4_Sb_1.6_Te_3_ and (e) Bi_0.4_Sb_1.6_Te_3_ + 0.5 wt.% SnSe. Insets: corresponding energy‐dispersive X‐ray spectroscopy (EDS) elemental maps. (f) Electron backscatter diffraction (EBSD) inverse pole figure (IPF) map of pristine Bi_0.4_Sb_1.6_Te_3_ and (g) the corresponding grain‐size distribution. (h) EBSD IPF map of Bi_0.4_Sb_1.6_Te_3_ + 0.5 wt.% SnSe and (i) the corresponding grain‐size distribution.

To elucidate the influence of SnSe incorporation on the microstructure of Bi_0.4_Sb_1.6_Te_3_, electron backscatter diffraction (EBSD) was performed on both the pristine and SnSe‐containing samples. As shown in Figure 2f,g, the pristine Bi_0.4_Sb_1.6_Te_3_ exhibits a fine‐grained microstructure, with the majority of grains smaller than 5 µm and an average grain size of ∼2.42 µm. After incorporating 0.5 wt.% SnSe, the grain size distribution remains essentially unchanged, with most grains still below 5 µm and an average grain size of ∼2.79 µm (Figure 2h,i), indicating that SnSe incorporation does not induce appreciable grain growth. The inverse pole figure (IPF) maps and the corresponding texture analysis are presented in Figures S6–S8. Both samples exhibit a pronounced (00*l*) preferred orientation, indicating that the crystallographic texture is well preserved after SnSe incorporation. Consequently, the excellent microstructural stability of the Bi_0.4_Sb_1.6_Te_3_ matrix is maintained despite the introduction of SnSe. These observations suggest that the changes in thermoelectric transport properties cannot be attributed to grain coarsening or texture evolution. Instead, they are more likely associated with the interfacial chemical reconstruction and compositional redistribution induced by SnSe, as discussed in the following microscopic analyses.

To gain deeper insight into the nanoscale characteristics of the SnSe‐induced interfacial reconstruction, a cross‐sectional lamella of Bi_0.4_Sb_1.6_Te_3_ + 0.5 wt.% SnSe was prepared by focused ion beam (FIB) milling and subsequently characterized using transmission electron microscopy (TEM). Figure 3a shows a low‐magnification scanning TEM (STEM) image together with the corresponding EDS elemental maps of Bi, Sb, Te, Sn, and Se. A distinct interfacial transition layer is observed between the Bi_0.4_Sb_1.6_Te_3_ matrix and the SnSe phase, where all five elements are simultaneously distributed, indicating the formation of a chemically intermixed interface rather than an abrupt phase boundary. The chemically mixed interfacial transition region exhibits a typical thickness of approximately 1.5–2.0 µm, as estimated from the low‐magnification STEM image and corresponding EDS elemental distribution. Notably, the Bi concentration decreases in the matrix adjacent to the interface, suggesting the migration of Bi toward the interfacial transition layer during sintering (Figures S9–S11). The low‐magnification TEM image in Figure 3b further reveals the interfacial region between the Bi_0.4_Sb_1.6_Te_3_ matrix and the transition layer, while the enlarged image in Figure 3c demonstrates a smooth and continuous interface. As shown in the high‐resolution TEM (HRTEM) image (Figure 3d), the transition layer exhibits an amorphous structure, providing direct evidence of SnSe‐induced interfacial chemical reconstruction. Such an amorphous interfacial layer is expected to introduce pronounced structural disorder and a high intensity of heterogeneous interfaces, thereby strengthening phonon scattering and suppressing the *κ*_l_. Figure 3e presents a magnified HRTEM image of the Bi_0.4_Sb_1.6_Te_3_ matrix adjacent to the reconstructed interface (highlighted in Figure 3d), where pronounced local lattice distortion is clearly observed. The corresponding selected‐area electron diffraction (SAED) pattern (Figure 3f) confirms that the Bi_0.4_Sb_1.6_Te_3_ matrix is oriented along the [120] zone axis.

**FIGURE 3:**
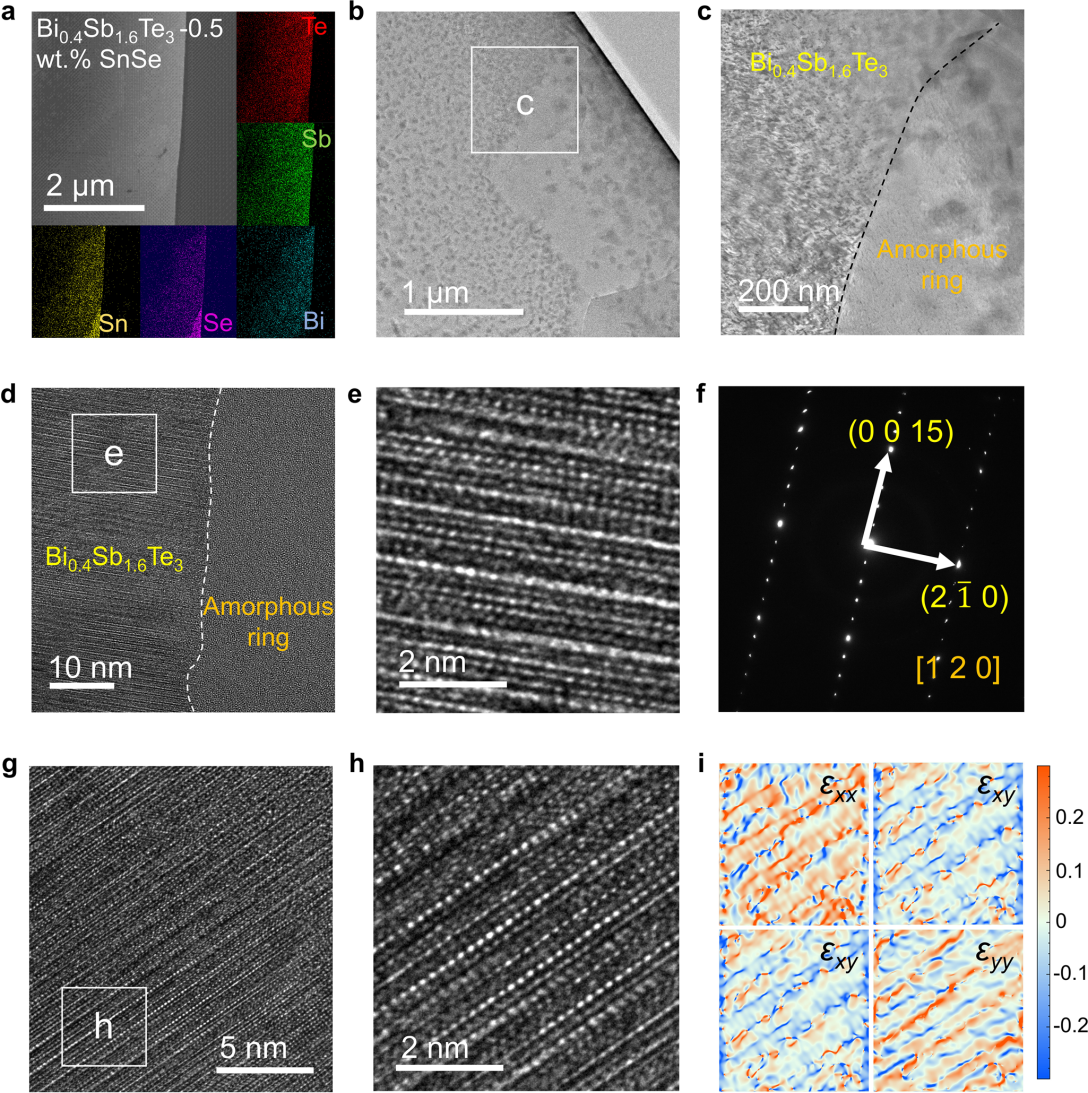
Microstructural and nanostructural characterization of Bi_0.4_Sb_1.6_Te_3_ + 0.5 wt.% SnSe. (a) Low‐magnification scanning transmission electron microscopy (STEM) image and the corresponding EDS elemental maps. (b) Low‐magnification transmission electron microscopy (TEM) image of the interfacial region between the Bi_0.4_Sb_1.6_Te_3_ matrix and the transition layer. (c) Enlarged TEM image of the region highlighted in (b). (d) High‐resolution TEM (HRTEM) image of the reconstructed interface. (e) Enlarged HRTEM image of the Bi_0.4_Sb_1.6_Te_3_ matrix adjacent to the interface, extracted from (d). (f) Corresponding selected‐area electron diffraction (SAED) pattern acquired along the [120] zone axis. (g) HRTEM image of the Bi_0.4_Sb_1.6_Te_3_ matrix away from the reconstructed interface. (h) Enlarged HRTEM image of the region highlighted in (g). (i) Corresponding strain maps derived from (h) along different directions.

To examine the structural evolution beyond the reconstructed interface, another region within the Bi_0.4_Sb_1.6_Te_3_ matrix was investigated. As shown in Figure 3g,h, well‐resolved lattice fringes are preserved, while pronounced local lattice distortion is still evident. The corresponding strain maps (Figure 3i) reveal significant lattice bending accompanied by multiaxial strain fluctuations, which are likely induced by the compositional redistribution and the formation of Sb‐rich precipitates within the surrounding matrix. These results indicate that the influence of SnSe extends beyond the immediate interfacial region. In addition to inducing interfacial chemical reconstruction, SnSe incorporation generates localized lattice imperfections and heterogeneous strain fields throughout the Bi_0.4_Sb_1.6_Te_3_ matrix. Such structural features provide abundant phonon‐scattering centers, thereby effectively suppressing the *κ*_l_.

### 2.2 Thermoelectric Performance of Bi_0.4_Sb_1.6_Te_3_ Incorporated With SnSe

To evaluate the effect of SnSe‐induced interfacial reconstruction on the thermoelectric transport properties, the temperature‐dependent transport performance of Bi_0.4_Sb_1.6_Te_3_ + *x* wt.% SnSe (*x* = 0, 0.25, 0.5, 0.75, 1, and 1.5) was measured over the temperature range of 303–423 K. Owing to the pronounced crystallographic texture of the spark plasma‐sintered samples, all transport properties were measured along the in‐plane direction, perpendicular to the pressing direction. Figure 4a presents the temperature dependence of the *S*. All samples exhibit positive *S* values throughout the measured temperature range, confirming their p‐type conduction behavior. The magnitude of *S* gradually decreases with increasing SnSe content, indicating an increase in the hole carrier concentration (*n*_h_), which is further corroborated by the Hall measurements discussed below. As shown in Figure 4b, the *σ* increases monotonically with increasing SnSe content, rising from 728.2 S cm^−1^ for pristine Bi_0.4_Sb_1.6_Te_3_ to 1883.6 S cm^−1^ for Bi_0.4_Sb_1.6_Te_3_ + 1.5 wt.% SnSe at 303 K. This remarkable enhancement is closely associated with the SnSe‐induced interfacial reconstruction, which effectively regulates the carrier transport behavior. The underlying transport mechanism is further elucidated through Hall‐effect measurements and single parabolic band (SPB) analysis [49, 50] in the following section. Although the incorporation of SnSe leads to a gradual reduction in *S*, the substantial increase in *σ* more than compensates for this decrease, resulting in a maximum power factor (*S* ^2^ *σ*) 37.4 µW cm^−1^ K^−2^ at 0.5 wt.% SnSe as shown in Figure 4c.

**FIGURE 4:**
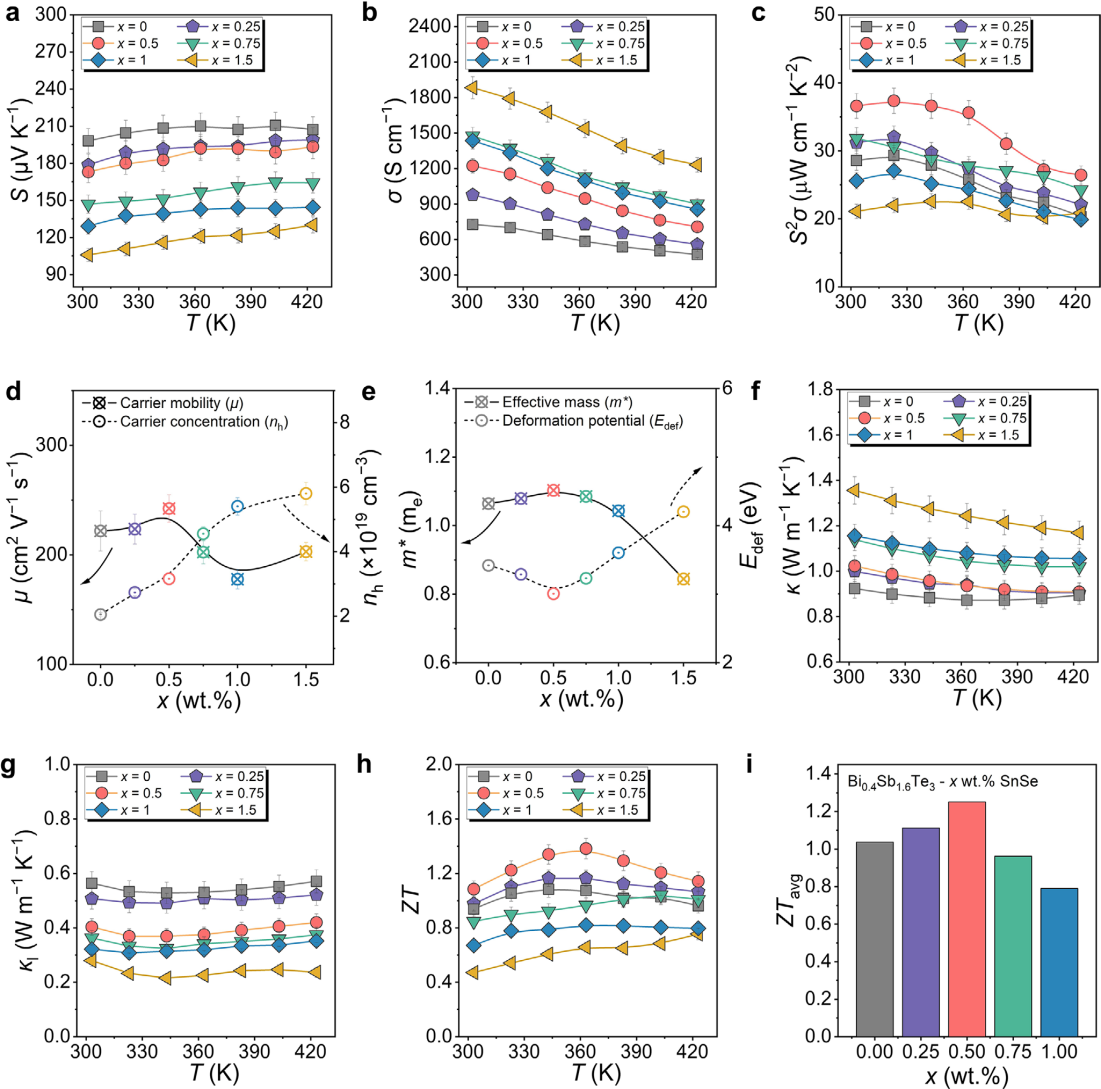
Thermoelectric transport properties of Bi_0.4_Sb_1.6_Te_3_ + *x* wt.% SnSe (*x* = 0, 0.25, 0.5, 0.75, 1, and 1.5 ). Temperature‐dependent (a) Seebeck coefficient (*S*), (b) electrical conductivity (*σ*), and (c) power factor (*S* ^2^ *σ*). (d) Room‐temperature hole carrier concentration (*n*_h_) and carrier mobility (*µ*) at room temperature. (e) Calculated room‐temperature effective mass (m*) and deformation potential (*E*_def_) estimated using the single parabolic band (SPB) model. (f) Total thermal conductivity (*κ*), (g) lattice thermal conductivity (*κ*_l_), and (h) *ZT* as a function of temperature. (i) Average *ZT* (*ZT*_avg_) value as a function of SnSe content.

To elucidate the origin of the electrical transport behavior, Hall‐effect measurements were performed to determine the *n*_h_ and *µ* of all samples, as shown in Figure 4d. With increasing SnSe content, the *n*_h_ continuously increases, which is the primary origin of the enhanced *σ*. In contrast, the *µ* exhibits a non‐monotonic dependence on SnSe content. At low SnSe contents (0–0.5 wt.%), interfacial reconstruction is associated with relatively favorable carrier transport. However, with further SnSe incorporation, excessive interfacial reconstruction introduces additional structural disorder, amorphous interfacial regions, lattice distortion, and interfacial defects, leading to enhanced carrier scattering and a decrease in weighted mobility (*µ*_w_) (Figure S12). Therefore, the Sb‐rich precipitates should be regarded as a structural consequence of SnSe‐induced interfacial reconstruction rather than as a direct origin of enhanced *µ*. Consequently, the continuous increase in *σ* at high SnSe contents is primarily governed by the increased *n*_h_, whereas the reduced *µ*_w_ indicates a deterioration in intrinsic transport quality due to enhanced structural and interfacial scattering.

To gain further insight into the origin of the increased *n*_h_, first‐principles density functional theory (DFT) calculations were carried out to compare the electronic structures of pristine and Bi‐deficient Bi_0.4_Sb_1.6_Te_3_ (Figure S13). The calculated bandgap decreases from 0.0455 eV for pristine Bi_0.4_Sb_1.6_Te_3_ to 0.0352 eV for the Bi‐deficient model. This slight bandgap narrowing may facilitate carrier excitation and contribute to the increased *σ*. However, it is unlikely to be the sole origin of the experimentally observed increase in *n*_h_. Instead, the increased *n*_h_ is more reasonably attributed to SnSe‐induced compositional redistribution and defect‐chemistry modulation. In particular, Bi migration toward the reconstructed interface can create local Bi deficiency in the adjacent Bi_0.4_Sb_1.6_Te_3_ matrix, which may promote acceptor‐type defect formation and increase the *n*_h_. This interpretation is consistent with the reduced *S* and enhanced *σ* observed experimentally. To further clarify the driving force for Bi redistribution, the thermodynamic stability of Bi at different positions across the Bi_0.4_Sb_1.6_Te_3_/SnSe interface was evaluated. The calculated relative energies follow the relationship Δ*E*_interface_ (−0.0508 eV) < 0< Δ*E*_SnSe_ (0.1004 eV), indicating that Bi is thermodynamically more stable at the interfacial region than within the SnSe lattice. This prediction is fully consistent with the STEM‐EDS results, which reveal Bi depletion in the adjacent Bi_0.4_Sb_1.6_Te_3_ matrix together with Bi enrichment within the chemically intermixed amorphous transition layer. Therefore, the DFT calculations provide compelling theoretical evidence for the proposed SnSe‐induced interfacial reconstruction mechanism, whereby Bi preferentially migrates toward and accumulates at the reconstructed interface rather than directly diffusing into the SnSe phase.

To further elucidate the evolution of *n*_h_ and *µ*, the SPB model was employed to estimate the effective mass (m*) and deformation potential (*E*_def_) as a function of SnSe content (Figure 4e). As the SnSe content increases from 0 to 0.5 wt.%, m* changes only slightly from 1.06 m_e_ to 1.10 m_e_, suggesting only limited variation in the density‐of‐states m* within this composition range. In contrast, *E*_def_ decreases over the same composition range, indicating a reduced apparent carrier‐scattering strength within the assumptions of the acoustic‐phonon‐scattering SPB model. This trend is consistent with the initially enhanced *µ*. With further increasing SnSe content from 0.5 to 1.5 wt.%, m* gradually decreases from 1.10 m_e_ to 0.84 m_e_, whereas *E*_def_ increases continuously. The increase in *E*_def_ reflects stronger effective carrier scattering, which may be associated with the increased structural disorder, lattice distortion, and interfacial defects introduced by excessive interfacial reconstruction. Because *E*_def_ is derived from a simplified SPB model, its variation is interpreted here as a qualitative indicator of changes in carrier‐scattering strength rather than a direct measurement of carrier‐lattice coupling. Overall, within the SPB framework, the evolution of *µ* appears to be more closely associated with changes in the effective scattering parameter than with large variations in m*. Accordingly, moderate SnSe‐induced interfacial reconstruction is associated with improved carrier transport, whereas excessive reconstruction introduces additional scattering centers and deteriorates the intrinsic carrier transport quality.

To investigate the influence of SnSe‐induced interfacial reconstruction on thermal transport, the in‐plane thermal diffusivity (*D*) of Bi_0.4_Sb_1.6_Te_3_ + *x* wt.% SnSe (*x* = 0, 0.25, 0.5, 0.75, 1, and 1.5) was measured, as shown in Figure S14. The *κ* was calculated according to *κ* = *DρC*_p_, where *C*_p_ is the specific heat capacity and *ρ* is the mass density. Figure 4f presents the temperature dependence of *κ* over the range of 303–423 K. With increasing SnSe content, *κ* gradually increases, primarily due to the enhanced *κ*_e_ resulting from the substantial increase in *σ*. To distinguish the electronic and lattice contributions, *κ*_e_ was estimated using the Wiedemann‐Franz relation (*κ*_e_ = *LσT*), where the Lorenz number (*L*) was determined from the SPB model (Figures S14 and S15) [51, 52]. The *κ*_l_ was subsequently obtained by subtracting *κ*_e_ from *κ*. As shown in Figure 4g, *κ*_l_ decreases monotonically with increasing SnSe content, demonstrating that SnSe‐induced interfacial reconstruction effectively suppresses lattice heat transport. To further elucidate the origin of the reduced *κ*_l_, the Debye–Callaway model was employed to analyze the phonon‐scattering mechanisms (Figure S16) [53]. For pristine Bi_0.4_Sb_1.6_Te_3_, lattice thermal transport is predominantly governed by Umklapp, grain‐boundary, point‐defect, and dislocation scattering, resulting in a *κ*_l_ of approximately 0.56 W m^−1^ K^−1^. Following SnSe incorporation, the reconstructed amorphous interfacial regions, Sb‐rich nanoprecipitates, lattice distortion, and interfacial defects collectively establish a hierarchical phonon‐scattering network. The synergistic effect of these multiscale scattering centers markedly suppresses phonon transport, leading to the continuous reduction in *κ*_l_. Benefiting from the simultaneous enhancement of the *S* ^2^ *σ* and suppression of the *κ*_l_, the optimized Bi_0.4_Sb_1.6_Te_3_ + 0.5 wt.% SnSe composite achieves a maximum *ZT* of ∼1.38 at 363 K (Figure 4h) and an average *ZT* (*ZT*_avg_) of ∼1.25 over the temperature range of 303–423 K (Figure 4i).

### 2.3 Device Performance of Single‐Leg Module Fabricated by Optimized Bi_0.4_Sb_1.6_Te_3_

To evaluate the practical performance of the optimized Bi_0.4_Sb_1.6_Te_3_ material, a single‐leg TED was designed through a combination of finite‐element simulations and experimental validation (Figure S17). Figure 5a–d presents the ANSYS Multiphysics simulations of the *η* as a function of the geometric dimensions of the thermoelectric leg, including its height, width, and length. The simulations indicate that the highest *η* is achieved with an optimized leg dimension of 3.3 × 3.3 × 5.5 mm^3^. Figure 5e and Figure S18 show the simulated temperature distribution across the thermoelectric leg under a Δ*T* of 200 K. A nearly uniform temperature distribution is obtained, indicating that the Ni diffusion barrier effectively minimizes interfacial thermal resistance and promotes efficient heat transfer between the Bi_0.4_Sb_1.6_Te_3_ leg and the Cu electrodes. Based on the optimized simulation results, a single‐leg TED with the optimized dimensions (3.3 × 3.3 × 5.5 mm^3^) was fabricated using the optimized Bi_0.4_Sb_1.6_Te_3_ material, with Ni serving as the diffusion barrier layer and Cu as the electrode. Figure 5f,g shows the output voltage (*U*) and output power (*P*) as functions of the current (*I*) under different Δ*T*s. The maximum *P* reaches 0.03 W at 1.81 A when Δ*T* = 200 K. Figure 5h presents the heat flow (*Q*) as a function of *I* under different Δ*T* values. As expected, *Q* increases with increasing *I*, consistent with the electrical and thermal transport characteristics of the device. Consequently, the fabricated single‐leg device achieves a peak *η* of ∼6.47% at Δ*T* = 200 K, as shown in Figure 5i, in excellent agreement with the simulated value of 6.78%, demonstrating the reliability of the finite‐element model for device design and performance prediction. The long‐term interfacial stability of the device was further evaluated by combining thermal cycling tests with cross‐sectional SEM characterization. As shown in Figure S19, the cross‐sectional SEM image together with the corresponding elemental maps and line‐scan profiles reveals a well‐preserved interface after thermal cycling. No discernible voids, cracks, or elemental interdiffusion are observed at the hot‐side contact, confirming the excellent interfacial integrity between the Bi_0.4_Sb_1.6_Te_3_ leg, the Ni diffusion barrier, and the Cu electrode. These results demonstrate the excellent structural stability and reliability of the fabricated TED for practical energy‐conversion applications.

**FIGURE 5:**
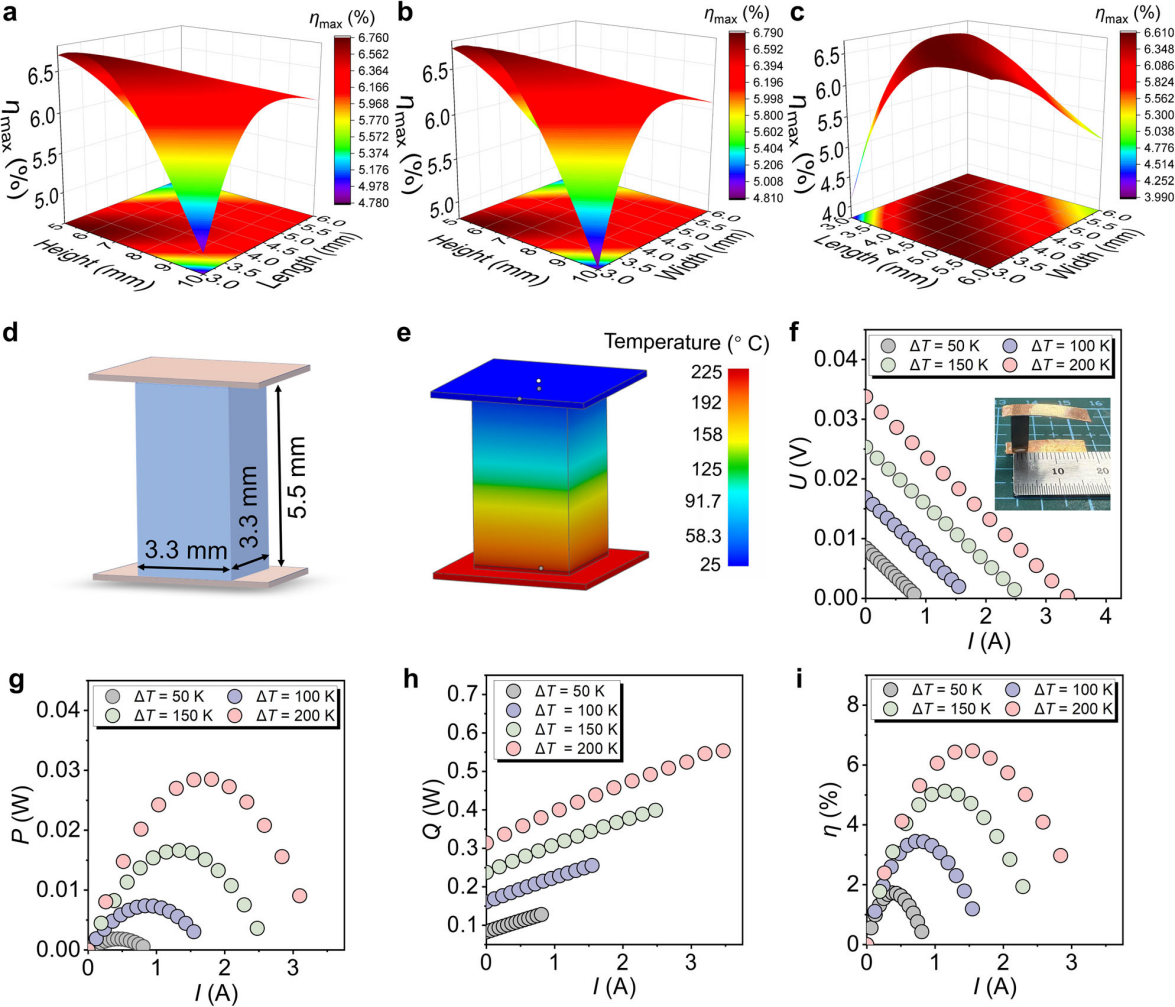
Design, simulation, and thermoelectric performance of the single‐leg device. (a–c) Finite‐element simulations of the *η* as a function of the leg dimensions (height, width, and length). (d) Schematic illustration of the optimized single‐leg TED. (e) Simulated temperature distribution across the optimized device under a temperature difference (Δ*T*) of 200 K. (f) Measured output voltage (*U*) as a function of current (*I*) under different Δ*T*s. Inset: Photograph of the fabricated single‐leg TED. Measured (g) output power (*P*), (h) heat flow (*Q*), and (i) *η* as functions of *I* under different Δ*T* values.

## 3 Conclusion

In summary, we have demonstrated that SnSe serves as an effective interfacial reconstruction agent for enhancing the thermoelectric performance of Bi_0.4_Sb_1.6_Te_3_. The incorporation of SnSe triggers interfacial chemical reconstruction at the Bi_0.4_Sb_1.6_Te_3_/SnSe interface, accompanied by Bi redistribution, the formation of Sb‐rich precipitates, and the development of chemically intermixed amorphous interfacial regions. The reconstructed interface promotes carrier transport at moderate SnSe contents, while the associated amorphous interfacial regions, lattice distortion, and interfacial defects establish a hierarchical phonon‐scattering network that effectively suppresses the *κ*_l_. Consequently, the optimized Bi_0.4_Sb_1.6_Te_3_ + 0.5 wt.% SnSe composite achieves a maximum *ZT* of ∼1.38 at 363 K and a *ZT*_avg_ of ∼1.25 over the temperature range of 303–423 K. To demonstrate its practical applicability, a single‐leg TED was designed through a combination of finite‐element simulations and experimental optimization. The optimized device delivers a high *η* of ∼6.47% under a Δ*T* of 200 K, highlighting the excellent device‐level performance of the optimized material. Overall, this work establishes interfacial reconstruction as an effective strategy for the synergistic regulation of carrier and phonon transport, providing a general design principle for developing high‐performance thermoelectric materials and efficient energy‐conversion devices.

## Author Contributions

**Wenyi Chen**: methodology, visualization, writing – original draft, investigation, conceptualization, software. **Xiao‐Lei Shi**: conceptualization, data curation, supervision, resources, project administration, formal analysis, validation, investigation, funding acquisition, writing – review and editing. **Meng Li**: software, formal analysis. **Chao Zhang**: methodology, formal analysis. **Konstantin Faershteyn**: methodology, formal analysis. **Siqi Liu**: software, methodology. **Yongqi Chen**: formal analysis. **Chengjiong He**: formal analysis. **Wanyu Lyu**: resources. **Jun Pei**: software. **Nanhai Li**: formal analysis. **Zhi‐Gang Chen**: conceptualization, data curation, supervision, resources, project administration, formal analysis, validation, writing – review and editing, investigation, funding acquisition.

## Funding

This work was financially supported by the Australian Research Council, HBIS‐UQ Innovation Centre for Sustainable Steel project, and QUT Capacity Building Professor Program.

## Conflicts of Interest

The authors declare no conflicts of interest.

## Supporting information




**Supporting File**: advs78266‐sup‐0001‐SuppMat.docx.

## Data Availability

The data that support the findings of this study are available from the corresponding author upon reasonable request.
