# Supplementary material for: Interfacial Reconstruction Enables Enhanced Thermoelectric Performance in Bismuth Telluride‐Based Alloys
Source: Adv Sci (Weinh). 2026 Oct 15:e78266. Online ahead of print. doi: 10.1002/advs.78266 (PMC13672693; doi:10.1002/advs.78266)
Supplement: Supplementary file 1 [file ADVS-9999-e78266-s001.docx]

**Supporting information**

Interfacial Reconstruction Enables Enhanced Thermoelectric Performance in Bismuth Telluride-Based Alloys

*Wenyi Chen,^1^ Xiao-Lei Shi,^1,*^ Meng Li,^1^ Chao Zhang,^2^ Konstantin Faershteyn,^2^ Siqi Liu,^1^ Yongqi Chen,^1^ Chengjiong He,^1^ Wanyu Lyu,^1^ Jun Pei,^1^ Nanhai Li,^1^ Zhi-Gang Chen^1,*^*

^1^ ARC Research Hub in Zero-emission Power Generation for Carbon Neutrality, Centre for Materials Science, and School of Chemistry and Physics, Queensland University of Technology, Brisbane, Queensland 4000, Australia;

^2^ Central Analytical Research Facility, Institute for Future Environments, Queensland University of Technology, Brisbane, Queensland 4000, Australia.

**Corresponding Author**

* Xiao-Lei Shi: [xiaolei.shi@qut.edu.au](mailto:xiaolei.shi@qut.edu.au);

* Zhi-Gang Chen: [zhigang.chen@qut.edu.au](mailto:zhigang.chen@qut.edu.au).

1. **Experimental Section**

*Chemicals*: Bismuth nitrate pentahydrate (Bi(NO_3_)_3_·5H_2_O, 99.99 %, Sigma-Aldrich), sodium tellurite (Na_2_TeO_3_, 99.9 %, Sigma-Aldrich), antimony oxide (Sb_2_O_3_, 99.9 %, Sigma-Aldrich), tin chloride dihydrate (SnCl_2_·2H_2_O, 99.9 %, Sigma-Aldrich), sodium selenite (Na_2_SeO_3_, 99.9 %, Sigma-Aldrich), ethylene glycol (EG, 99 %, Sigma-Aldrich), and sodium hydroxide (NaOH, 96 %, Sigma-Aldrich) were used for solvothermal synthesis without further purification.

*Materials synthesis and preparation*: Bi(NO_3_)_3_·5H_2_O (1.21 mmol), Na_2_TeO_3_ (9.08 mmol), and Sb_2_O_3_ (2.42 mmol) were dissolved into EG (36 ml) with 4 ml NaOH (5 mol L^–1^) to form the precursor solution, kept stirring until obtaining the clear solution at room temperature. Similarly, SnCl_2_·2H_2_O (10.1 mmol) and Na_2_SeO_3_ (10.1 mmol) was dissolved in 40 ml EG with NaOH (10 mol L^–1^) to form the precursor solution for SnSe, kept stirring until obtaining the clear solution at room temperature. The two prepared solutions were then separately sealed in two 125 ml polytetrafluoroethylene-lined stainless-steel autoclaves. The autoclaves were heated in an oven at 230 °C for 24 h, followed by natural cooling to room temperature. After the synthesis, the two types of products were cooled to room temperature naturally and then collected by centrifugation and washed with deionized water and ethanol several times. Finally, the washed products were dried in the vacuum oven at 60 °C for 24 h to avoid oxidation.

The synthesized SnSe microplates were ground into finer powders using an agate mortar and then mixed uniformly with synthesized Bi_0.4_Sb_1.6_Te_3_. The prepared powders of Bi_0.4_Sb_1.6_Te_3_ + *x* wt.% SnSe (*x* = 0, 0.25, 0.5, 0.75, 1, 1.5) were sintered into pellets using spark plasma sintering (SUGA SPSS2200 system) with 50 MPa at 723 K for 5 min, using a graphite die with a 12.7 mm diameter.

*Characterization*: The microstructure of as-synthesized Bi_0.4_Sb_1.6_Te_3_ + *x* wt.% SnSe (*x* = 0, 0.25, 0.5, 0.75, 1, 1.5) pellets were prepared for microstructural analysis using standard mechanical grinding and polishing techniques. Microstructure and grain information were characterized through scanning electron microscope (SEM, Hitachi SU7000) and electron backscatter diffraction (equipped in Tescan S8000X Plasma), respectively. The nanostructure of the sample was characterized through TEM (JEOL ARM200F NeoARM). The element distribution and the chemical composition of the pellets were determined through backscattered electron (BSE) imaging and energy-dispersive X-ray spectroscopy (EDS) elemental mapping (equipped in Hitachi SU7000 and JEOL ARM200F NeoARM). The phase information and crystal lattice structure of the samples were determined by Rigaku SmartLab diffractometer equipped with CuKα radiation over an angular range of 10° to 80° in 0.02° increments.

*Thermoelectric property measurement*: The Hall coefficient (*R*_H_) was determined using the van der Pauw method on a Hall measurement system (DX-50, XIAMEN DEXING MAGNET TECH CO., LTD., China) under a reversible magnetic field of 0.5 T at room temperature. The Hall carrier concentration (*n*_H_) was calculated using the following relation: *n*_H_ = 1/(*eR*_H_), where *e* is the elementary charge. The Hall mobility (*μ*_H_) was obtained from the relation: *μ*_H_ = *σR*_H_, where *σ* is the electrical conductivity measured at room temperature. Ulvac-Riko ZEM-3 was used to measure the Seebeck coefficient (*S*) and electrical conductivity (*σ*) of all samples from 303 K to 423 K. The thermal conductivity (*κ*) values were calculated by the formula *κ* =*D*×*C*_p_×*ρ*,^[1]^ where *D* is the thermal diffusivity, *C*_p_ is the specific heat capacity, and *ρ* is the sample density (measured through Archimedes method). *D* was measured by a laser flash method (Netzsch LFA 467 system), the samples were sintered as the big bulk and then sectioned into (10 × 10 × 1.3 mm) to measure the in-plane *D*. The electronic thermal conductivity (*κ*_e_) was calculated using Wiedemann–Franz law: *κ*_e_=*LσT*,^[1]^ where *L* is the Lorenz number, calculated based on the single parabolic band (SPB) model, and *T* is the absolute temperature. The lattice thermal conductivity (*κ*_l_) was calculated using *κ*_l_ = *κ*– *κ*_e_.

*Finite element simulation*: Finite element simulations were performed using the ANSYS Workbench platform. To improve the accuracy and fidelity of the results, a multiphysics coupling approach was employed to simultaneously simulate both thermal and electrical fields. The temperature-dependent *S*, *σ*, and *κ* for the thermoelectric materials (Bi_0.4_Sb_1.6_Te_3_ + 0.5 wt.% SnSe) were defined based on experimentally measured data. For geometric optimization, the ANSYS DesignXplorer module was integrated with a Design of Experiments (DOE) approach to identify the optimal leg geometry with respect to power output and thermoelectric conversion efficiency.

The governing equation used in the simulation is:

$\nabla(\kappa\nabla T)+\frac{\boldsymbol{J}^{2}}{\sigma}-T\boldsymbol{J}\cdot[(\frac{\partial S}{\partial T})\nabla T+(\nabla S)_{T}]=0$	(S1-1)

$\nabla\cdot\boldsymbol{J}=0$	(S1-2)

$\boldsymbol{J}=-\sigma(\nabla V+S\nabla T)$	(S1-3)

$\boldsymbol{q}=ST\boldsymbol{J}-\kappa\nabla T$	(S1-4)

In the above equations, ***J*** and ***q*** represent the electric current density and heat flux density, respectively. *V* is the electric potential. All transport properties *S*, *κ*, and *σ* are considered temperature dependent.

Thermoelectric output power and conversion efficiency:

$P=I\cdot V_{load}$	(S1-5)

$\eta= \frac{P}{Q_{in}}$	(S1-6)

where *P* is output power, and *Q*_in_ is the heat input at hot side.

To simplify the model and reduce computational complexity, the following assumptions were made:

(1) Heat radiation is neglected.
(2) Heat convection is included, with a fixed convection coefficient of 5 W·m⁻^2^·K⁻^1^ for air;
(3) A constant nominal value for both electrical and thermal contact resistance is applied at all interfaces, including the leg/solder interface, with no temperature dependence.
(4) Heat sinks are not explicitly modelled; their effect is incorporated through thermal boundary conditions.
(5) Nonlinear temperature dependence of thermoelectric properties is fully considered, based on experimental measurements.

Barrier layer is Nickel and electrodes is copper. The corresponding electrical and thermal properties are sourced from the ANSYS material library.

*Computational calculation*: First-principles density functional theory (DFT) calculations were performed using the projector-augmented wave (PAW) method as implemented in the Vienna ab initio Simulation Package (VASP).^[2-6]^ The exchange–correlation interaction was described by the Perdew–Burke–Ernzerhof generalized gradient approximation (GGA-PBE) functional.^[7]^ A Γ-centered k-point mesh with a reciprocal-space sampling density of 2π×0.03 Å^−1^ was employed for self-consistency. The plane-wave basis set was expanded with a kinetic energy cutoff of 450 eV. The plane-wave basis set was expanded with a kinetic energy cutoff of 450 eV. Structural relaxations were performed until the electronic energy converged to 1×10^−6^ eV and the residual force on each atom was less than 0.01 eV/Å. Spin–orbit coupling (SOC) was included in all calculations as Bi and Te are heavy elements.^[8]^ Spin–orbit coupling (SOC) was included in all calculations as Bi and Te are heavy elements.

To construct the Bi_0.4_Sb_1.6_Te_3_/SnSe heterostructure, the (100) surfaces of Bi_0.4_Sb_1.6_Te_3_ and SnSe were cleaved and expanded into commensurate supercells to minimize the lattice mismatch. The optimized supercells were then combined to form the interface, and the interface energy was subsequently evaluated based on the relaxed heterostructure model.^[9]^

*Single parabolic band (SPB) modelling:* There are:^[10-13]^

$S\left( \eta\right)=\frac{k_{B}}{e}\cdot\left[ \frac{\left( r+\frac{5}{2} \right)\cdot F_{r+\frac{3}{2}}\left( \eta\right)}{\left( r+\frac{3}{2} \right)\cdot F_{r+\frac{1}{2}}\left( \eta\right)}-\eta\right]$	(S1-7)

$p=\frac{1}{e\cdot R_{H}}=\frac{{(2m^{*}\cdot k_{B}T)}^{\frac{3}{2}}}{3\pi^{2}\hbar^{3}}\cdot\frac{\left( r+\frac{3}{2} \right)^{2}\cdot F_{r+\frac{1}{2}}^{2}(\eta)}{(2r+\frac{3}{2})\cdot F_{2r+\frac{1}{2}}(\eta)}$	(S1-8)

$\mu=\left[ \frac{e\pi\hbar^{4}}{\sqrt{2}{(k_{B}T)}^{\frac{3}{2}}}\frac{C_{l}}{{E_{def}}^{2}{(m^{*})}^{\frac{5}{2}}} \right]\frac{(2r+\frac{3}{2})\cdot F_{2r+\frac{1}{2}}(\eta)}{\left( r+\frac{3}{2} \right)^{2}\cdot F_{r+\frac{1}{2}}(\eta)}$	(S1-9)

$L={(\frac{k_{B}}{e})}^{2}\cdot\left\{ \frac{\left( r+\frac{7}{2} \right)\cdot F_{r+\frac{5}{2}}\left( \eta\right)}{\left( r+\frac{3}{2} \right)\cdot F_{r+\frac{1}{2}}\left( \eta\right)}-\left[ \frac{\left( r+\frac{5}{2} \right)\cdot F_{r+\frac{3}{2}}\left( \eta\right)}{\left( r+\frac{3}{2} \right)\cdot F_{r+\frac{1}{2}}\left( \eta\right)} \right]^{2} \right\}$	(S1-10)

where *η* is the reduced Fermi level, *k*_B_ is the Boltzmann constant, *r* is the carrier scattering factor (*r* = −1/2 for acoustic phonon scattering), *R_H_* is the Hall coefficient, *C_l_* is the elastic constant for longitudinal vibrations, and *E_def_* is the deformation potential coefficient, respectively. There is:^[10-13]^

*C_l_ = v*_L_*^2^⋅ d*	(S1-11)

The longitudinal velocity (*v*_L_) is derived from the reference ^[14]^. *F_i_(η)* is the Fermi integral, expressed as ^[10-13]^:

$F_{i}\left( \eta\right)=\int_{0}^{\infty} \frac{x^{i}}{1+e^{(x-\eta)}}dx$	(S1-12)

*Single-leg thermoelectric module fabrication and Measurement*: The single-leg module was fabricated by using SPS based on the high-performance Bi_0.4_Sb_1.6_Te_3_ + 0.5 wt.% SnSe. The sintered material with a 12.7 mm diameter and 1.4 cm height was using electroplating to deposit the Nickel (Ni) barrier layer on the materials. The deposited material was cleaned by deionized water to remove residual electroplating solution and then was sectioned into the leg with dimensions of **3.3 × 3.3 × 5.5 mm** using a diamond wire saw. Copper plates (0.5 mm thick) were subsequently bonded to the outer surfaces of the Ni layers to serve as electrodes. The electrical output and thermoelectric conversion efficiency (*η*) of the module were evaluated using a custom-built thermoelectric device measurement system. The measurement details can be found in previous work.

*Average ZT calculations*: The average *ZT* (*ZT*_avg_) value is given by:

${ZT}_{avg} = \frac{\int_{T_{c}}^{T_{h}} ZT\left( T \right) dT}{T_{h}- T_{c}}$	(S1-13)

where *T*_h_ and *T*_c_ are the applied temperatures on the thermoelectric materials at hot-side and cold-side temperatures, respectively.

*Weighted mobility* (*μ*_W_): Weighted mobility (*μ*_W_) can be given by the equation:^[15]^

$\mu_{W}=\frac{T^{-3/2}}{{300}^{-5/2}}\left[ \frac{exp\left[ \frac{\left| S \right|}{k_{B}/e}-2 \right]}{1 + exp\left[ -5\left( \frac{\left| S \right|}{k_{B}/e}-1 \right) \right]}+\frac{\frac{3}{\pi^{2}}\frac{\left| S \right|}{k_{B}/e}}{1 + exp\left[ 5\left( \frac{\left| S \right|}{k_{B}/e}-1 \right) \right]} \right]$	(S1-14)

*Debye-Callaway model calculation*: According to the classical Debye-Callaway model,^[16]^ the lattice thermal conductivity *κ*_l_ associated with the different phonon scattering mechanisms can be expressed as:

$\kappa_{l}=\frac{k_{B}}{2\pi^{2}v_{a}}\left( \frac{k_{B}T}{\hbar} \right)^{3}\int_{0}^{\frac{\theta_{D}}{T}} \frac{x^{4}e^{y}}{\tau^{-1}(e^{y}-1)^{2}}dy$	(S1-15)

where $\hbar$, $v_{a}$, and $\theta_{D}$ are the reduced Planck constant, average sound velocity, and Debye temperature, respectively. *y* = $\hbar$*ω*∕$k_{B}$T is the simplified phonon frequency, and *ω* is the phonon frequency. The overall phonon scattering relaxation time is expressed as:

$\tau^{-1}=\tau_{U}^{-1}+\tau_{GB}^{-1}+\tau_{NP}^{-1}+\tau_{PD}^{-1}+\tau_{D}^{-1}$	(S1-16)

Umklapp scattering (U) occurs when phonons within a crystal scatter off other phonons, and the relaxation time for this process follows a specific formula:

$\tau_{U}^{-1}=A_{N}\frac{h\gamma^{2}}{Mv_{a}^{2}}\omega^{2}Texp(-\frac{\theta_{D}}{3T})$	(S1-17)

The relaxation time for the Normal process is considered as an additional factor, represented by *A*N.

For common grain boundaries scattering (GB), the frequency-independent is given by:

$\tau_{GB}^{-1}=\frac{v}{d}$	(S1-18)

where *d* is the experimentally determined grain size.

The relaxation time of point defect scattering (PD) is calculated by:

$\tau_{PD}^{-1}=\frac{V\omega^{2}}{4\pi v_{a}^{3}}\Gamma$	(S1-19)

where *V* is the average atom volume, and *Γ* is the disorder parameter of mass field fluctuations (*Γ*_M_) and strain field fluctuations (*Γ*_S_).

Phonon scattering by point defects mainly comes from the combined effects of the mass and strain disorders, which can be quantitatively characterized by *Γ*_M_ and *Γ*s.^[17, 18]^ For the compound with the chemical composition X_1c1_X_2c2_X_3c3_…X_ncn_, the above two parameters are given by:^[17]^

*Γ = Γ*_M_ *+ Γ*_S_	(S1-20)

$\Gamma_{M} = \frac{\sum_{i=1}^{n} \left( \frac{\bar{M_{i}}}{\overset{̿}{M}} \right)^{2}f_{i}^{A}f_{i}^{B}\left( \frac{M_{i}^{A}-M_{i}^{B}}{\bar{M_{i}}} \right)^{2}}{\sum_{i=1}^{n} c_{i}}$	(S1-21)

$\Gamma_{S} = \frac{\sum_{i=1}^{n} {c_{i}\left( \frac{\bar{M_{i}}}{\overset{̿}{M}} \right)}^{2}f_{i}^{A}f_{i}^{B}\varepsilon_{i}\left( \frac{r_{i}^{A}-r_{i}^{B}}{\bar{r_{i}}} \right)^{2}}{\sum_{i=1}^{n} c_{i}}$	(S1-22)

where $\bar{\text{M}_{\text{i}}}$ is the average mass at the *i*^th^ site, $\overset{̿}{\text{M}}$ is the average mass per atom,$f_{i}^{A}$ $\text{f}_{\text{i}}^{\text{A}}$ and $\text{f}_{\text{i}}^{\text{B}}$ is the fractional occupancies for the atom A and B at the *i*^th^ site, $\text{M}_{\text{i}}^{\text{A}}$ and $\text{M}_{\text{i}}^{\text{B}}$ are the relative atomic mass of A and B, $\text{r}_{\text{i}}^{\text{A}}$ and $\text{r}_{\text{i}}^{\text{B}}$ are the atomic radius of each atoms, $\bar{\text{r}_{\text{i}}}$ is the average atomic radius. $\text{ε}_{\text{i}}$ is the parameter which relates to the Grueneisen parameter *γ* and some elastic constants. In this paper, $\text{ε}_{\text{i}}$ can be estimated by the equation as given below:^[19]^

$\varepsilon= \frac{2}{9}\left( \frac{6.4\gamma(1+v_{p})}{(1-v_{p})} \right)^{2}$	(S1-23)

where *v*_p_ the Poisson ratio, deriving from *v*_l_ and *v*_t_ sound velocities by the equation as:^[18, 20]^

$v_{p} = \frac{1-2\left( \frac{v_{t}}{v_{l}} \right)^{2}}{2-2\left( \frac{v_{t}}{v_{l}} \right)^{2}}$	(S1-24)

Gruneisen parameter (*γ*) has been calculated using Poisson ratio (*v*_p_) as:^[21]^

$\gamma= \frac{3}{2}\left( \frac{1-v_{p}}{2-3v_{p}} \right)$	(S1-25)

The strain field is represented by the dislocation scattering, which can be treated as two parts: dislocation cores and dislocation strain. The relaxation time with this process is represented in the equation:

Dislocation core:

$\tau_{DC}^{-1}= N_{D}\frac{\bar{V}^{\frac{4}{3}}}{v^{2}}\omega^{3}$	(S1-26)

Dislocation strain:

$\tau_{DS}^{-1}= 0.6B_{D}^{2}N_{D}\gamma^{2}\omega\left\{ \left\{ \frac{1}{2}+\frac{1}{24}{(\frac{1-2r}{1-r})}^{2}\left[ 1+{\sqrt{2}(\frac{v_{l}}{v_{t}})}^{2} \right]^{2} \right\} \right.$	(S1-27)

where $B_{D}$ is the effective Burger’s vector, $N_{D}$ is the dislocation density, *r* is the Poisson’s ratio, respectively.

The relaxation time influenced by precipitates can be simplified as:

$\tau_{NP}^{-1}=v\left\{ {(2\pi R^{2})}^{-1}+\left( \pi R^{2}\frac{4}{9}\left( \frac{\Delta Z}{Z} \right)^{2}\left( \frac{\omega R}{v} \right)^{4} \right)^{-1} \right\}N_{P}$	(S1-28)

*R* is the average diameter of the precipitates, *Z* is the matrix density, *∆Z* is the density difference between the matrix and precipitates, *N_p_* is the density of precipitates within the matrix.

**2. Supporting figures**

**Figure S1. (a)** Variation of Bi_0.4_Sb_1.6_Te_3_ lattice parameters as a function of SnSe content. Rietveld quantitative phase analysis of Bi_0.4_Sb_1.6_Te_3_ with **(b)** *x* = 0, **(c)** *x* = 0.25, **(d)** *x* = 0.5, **(e)** *x* = 0.75, and **(f)** *x* = 1 wt.% SnSe.

**Figure S2.** Characterizations of compositions of Bi_0.4_Sb_1.6_Te_3_ + 0.25 wt.% SnSe. **(a)** Backscattered electron (BSE) SEM images. **(b)** Corresponding energy dispersive X-ray spectroscopy (EDS) maps for Bi, Sb, Te, Sn, and Se.

**Figure S3.** Characterizations of compositions of Bi_0.4_Sb_1.6_Te_3_ + 0.75 wt.% SnSe. **(a)** Backscattered electron (BSE) SEM images. **(b)** Corresponding EDS maps for Bi, Sb, Te, Sn, and Se.

**Figure S4.** Characterizations of compositions of Bi_0.4_Sb_1.6_Te_3_ + 1 wt.% SnSe. **(a)** Backscattered electron (BSE) SEM images. **(b)** Corresponding EDS maps for Bi, Sb, Te, Sn, and Se.

**Figure S5.** Characterizations of compositions of Bi_0.4_Sb_1.6_Te_3_ + 1.5 wt.% SnSe. **(a)** Backscattered electron (BSE) SEM images. **(b)** Corresponding EDS maps for Bi, Sb, Te, Sn, and Se.

**Figure S6.** Electron backscatter diffraction (EBSD) Euler maps for **(a)** Bi_0.4_Sb_1.6_Te_3_ and **(b)** Bi_0.4_Sb_1.6_Te_3_ + 0.5 wt.% SnSe.

**Figure S7.** Electron backscatter diffraction (EBSD) analysis for inverse pole figure (IPF) maps of Bi_0.4_Sb_1.6_Te_3_ for **(a)** X, **(b)** Y, and **(c)** Z. **(d)** Corresponding texture analysis.

**Figure S8.** EBSD analysis for IPF maps of Bi_0.4_Sb_1.6_Te_3_ + 0.5 wt.% SnSe for **(a)** X, **(b)** Y, and **(c)** Z. **(d)** Corresponding texture analysis.

**Figure S9.** Characterizations of compositions of the focused ion beam sample taken from Bi_0.4_Sb_1.6_Te_3_ + 0.5 wt.% SnSe: scanning transmission electron microscopy (STEM) image and corresponding EDS maps for Bi, Sb, Te, Sn, and Se.

**Figure S10.** Characterizations of line scan for Bi_0.4_Sb_1.6_Te_3_ + 0.5 wt.% SnSe on the STEM image between different phases**.**

.

**Figure S11.** Characterizations of line scan for Bi_0.4_Sb_1.6_Te_3_ + 0.5 wt.% SnSe on the STEM image between different phases**.**

.

**Figure S12.** Temperature-dependent weighted mobility (*μ*_W_) of Bi_0.4_Sb_1.6_Te_3_ + *x* wt.% SnSe (*x* = 0, 0.25, 0.5, 0.75, 1, 1.5).

**Figure S13.** Calculated band structures and DOSs through DFT calculations: Band structures and DOS of **(a)** pristine Bi_0.4_Sb_1.6_Te_3_ and **(b)** Bi-deficient Bi_0.4_Sb_1.6_Te_3_.

.

**Figure S14.** Critical properties for evaluating the thermoelectric performance of Bi_0.4_Sb_1.6_Te_3_ + *x* wt.% SnSe (*x* = 0, 0.25, 0.5, 0.75, 1, 1.5): (**a**) Temperature-dependent Lorentz number (*L*). (**b**) Temperature-dependent thermal diffusivity (*D*)*.*

.

**Figure S15.** Temperature-dependent electronic thermal conductivity (*κ*_e_) of Bi_0.4_Sb_1.6_Te_3_ + *x* wt.% SnSe (*x* = 0, 0.25, 0.5, 0.75, 1, 1.5).

**Figure S16. (a)** Comparison of measured and calculated temperature-dependent lattice thermal conductivity (*κ*_l_) of pristine Bi_0.4_Sb_1.6_Te_3_ and Bi_0.4_Sb_1.6_Te_3_ + 0.5 wt.% SnSe (Calculation is based on Debye-Callaway model). **(b)** Frequency-dependent spectral lattice thermal conductivities (*κ*_s_) calculated by the Debye-Callaway model at room temperature. Here, U refers to the Umklapp process, PD refers to the point defect scattering, GB refers to grain boundary scattering, D refers to dislocation scattering, and NP refers to the nano precipitates.

**Figure S17.** Photograph of **(a)** Bi_0.4_Sb_1.6_Te_3_ + 0.5 wt.% SnSe composites sintered by spark plasma sintering (SPS) and **(b)** the thermoelectric leg sectioned through diamond wire saw.

**Figure S18.** Applying ANSYS for modelling of the single-leg module and corresponding simulated the temperature difference of the as-fabricated device. Environment temperature is 22° with heat convection of 5 W m^−2^ K^−1^.

.

**Figure S19. (a)** Cross-sectional SEM image of Bi_0.4_Sb_1.6_Te_3_ + 0.5 wt.% SnSe single leg with Ni diffusion barriers. Corresponding EDS elemental maps for **(b)** Ni, **(c)** Bi, **(d)** Sb, **(e)** Te, and **(f)** Sn and Se. **(g)** Line scan image of Bi_0.4_Sb_1.6_Te_3_ + 0.5 wt.% SnSe single leg with Ni diffusion barriers. **(h)** Corresponding EDS line scan profiles.

**Reference**

[1]	Y. Wang, W.-D. Liu, X.-L. Shi, M. Hong, L.-J. Wang, M. Li, H. Wang, J. Zou, Z.-G. Chen, Enhanced Thermoelectric Properties of Nanostructured n-Type Bi_2_Te_3_ by Suppressing Te Vacancy through Non-Equilibrium Fast Reaction, *Chem. Eng. J.* **2020**, *391*, 123513.

[2]	G. Kresse, J. Hafner, *Ab initio* molecular-dynamics simulation of the liquid-metal-amorphous-semiconductor transition in germanium, *Phys. Rev. B* **1994**, *49*, 14251.

[3]	G. Kresse, J. Furthmüller, Efficiency of *ab-initio* total energy calculations for metals and semiconductors using a plane-wave basis set, *Comp. Mater. Sci.* **1996**, *6*, 15.

[4]	G. Kresse, J. Hafner, Norm-conserving and ultrasoft pseudopotentials for first-row and transition elements, *J. Phys. Condens. Mat.* **1994**, *6*, 8245.

[5]	G. Kresse, J. Furthmüller, Efficient iterative schemes for *ab initio* total-energy calculations using a plane-wave basis set, *Phys. Rev. B* **1996**, *54*, 11169.

[6]	G. Kresse, D. Joubert, From ultrasoft pseudopotentials to the projector augmented-wave method, *Phys. Rev. B* **1999**, *59*, 1758.

[7]	J. P. Perdew, K. Burke, M. Ernzerhof, Generalized Gradient Approximation Made Simple, *Phys. Rev. Lett.* **1996**, *77*, 3865.

[8]	W. Setyawan, S. Curtarolo, High-throughput electronic band structure calculations: Challenges and tools, *Comp. Mater. Sci.* **2010**, *49*, 299.

[9]	Y. Qi, L. G. Hector, N. Ooi, J. B. Adams, A first principles study of adhesion and adhesive transfer at Al(111)/graphite(0001), *Surface Science* **2005**, *581*, 155.

[10]	X. L. Shi, K. Zheng, M. Hong, W. D. Liu, R. Moshwan, Y. Wang, X.-L. Qu, Z. G. Chen, J. Zou, Boosting the Thermoelectric Performance of p-Type Heavily Cu-Doped Polycrystalline SnSe *via* Inducing Intensive Crystal Imperfections and Defect Phonon Scattering, *Chem. Sci.* **2018**, *9*, 7376.

[11]	X. Shi, A. Wu, T. Feng, K. Zheng, W. Liu, Q. Sun, M. Hong, S. T. Pantelides, Z. G. Chen, J. Zou, High Thermoelectric Performance in p-type Polycrystalline Cd-doped SnSe Achieved by a Combination of Cation Vacancies and Localized Lattice Engineering, *Adv. Energy Mater.* **2019**, *9*, 1803242.

[12]	X. Shi, A. Wu, W. Liu, R. Moshwan, Y. Wang, Z.-G. Chen, J. Zou, Polycrystalline SnSe with Extraordinary Thermoelectric Property *via* Nanoporous Design, *ACS Nano* **2018**, *12*, 11417.

[13]	M. Jin, X.-L. Shi, T. Feng, W. Liu, H. Feng, S. T. Pantelides, J. Jiang, Y. Chen, Y. Du, J. Zou, Z.-G. Chen, Super Large Sn_1-_*_x_*Se Single Crystals with Excellent Thermoelectric Performance, *ACS Appl. Mater. Interfaces* **2019**, *11*, 8051.

[14]	K. Husnu, A. M. Mamedov, E. Ozbay, "Structural, elastic, and electronic properties of topological insulators: Sb_2_Te_3_ and Bi_2_Te_3_", presented at *2013 Joint IEEE International Symposium on Applications of Ferroelectric and Workshop on Piezoresponse Force Microscopy (ISAF/PFM)*, 21-25 July 2013, 2013.

[15]	G. J. Snyder, A. H. Snyder, M. Wood, R. Gurunathan, B. H. Snyder, C. Niu, Weighted Mobility, *Adv. Mater.* **2020**, *32*, 2001537.

[16]	J. Callaway, Model for lattice thermal conductivity at low temperatures, *Phys. Rev.* **1959**, *113*, 1046.

[17]	B. Abeles, Lattice Thermal Conductivity of Disordered Semiconductor Alloys at High Temperatures, *Phys. Rev.* **1963**, *131*, 1906.

[18]	J. Callaway, H. C. von Baeyer, Effect of Point Imperfections on Lattice Thermal Conductivity, *Phys. Rev.* **1960**, *120*, 1149.

[19]	C. Wan, W. Pan, Q. Xu, Y. Qin, J. Wang, Z. Qu, M. Fang, Effect of point defects on the thermal transport properties of (La*_x_*Gd_1−_*_x_*_)2_Zr_2_O_7_: Experiment and theoretical model, *Phys. Rev. B* **2006**, *74*, 144109.

[20]	D. S. Sanditov, V. N. Belomestnykh, Relation between the parameters of the elasticity theory and averaged bulk modulus of solids, *Tech. Phys+.* **2011**, *56*, 1619.

[21]	Y. Zhang, E. Skoug, J. Cain, V. Ozoliņš, D. Morelli, C. Wolverton, First-principles description of anomalously low lattice thermal conductivity in thermoelectric Cu-Sb-Se ternary semiconductors, *Phys. Rev. B* **2012**, *85*, 054306.
